# Down-regulated expression of CDK5RAP3 and UFM1 suggests a poor prognosis in gastric cancer patients

**DOI:** 10.3389/fonc.2022.927751

**Published:** 2022-10-27

**Authors:** Mi Lin, Ning-Zi Lian, Long-Long Cao, Chang-Ming Huang, Chao-Hui Zheng, Ping Li, Jian-Wei Xie, Jia-Bin Wang, Jun Lu, Qi-Yue Chen, Ya-Han Li, Zhu-Huai Peng, Xiao-Yu Zhang, Yi-Xian Mei, Jian-Xian Lin

**Affiliations:** ^1^ Department of Gastric Surgery, Fujian Medical University Union Hospital, Fuzhou, Fujian, China; ^2^ Key Laboratory of Ministry of Education of Gastrointestinal Cancer, Fujian Medical University, Fuzhou, Fujian, China; ^3^ Department of Gynecology, Fujian Obstetrics and Gynecology Hospital, Fuzhou, China; ^4^ The Union Clinical Medical College, Fujian Medical University, Fuzhou, Fujian, China

**Keywords:** gastric adenocarcinoma, CDK5RAP3, UFM1, AKT pathway, prognosis

## Abstract

**Purpose:**

The relationship between the CDK5RAP3 and UFM1 expression and the prolonged outcomes of patients who underwent gastric cancer (GC) surgery was investigated.

**Methods:**

Single-sample gene set enrichment analysis (ssGSEA), unsupervised clustering and other methods were used to verify the relationship between CDK5RAP3 and UFM1 in GC through public databases. Additionally, CDK5RAP3 and UFM1 expression in cancerous and paracancerous tissues of GC was analysed in the context of patient prognosis.

**Results:**

CDK5RAP3 and UFM1 expression was downregulated synchronously, the interaction was observed between the two proteins, and UFM1 and CDK5RAP3 expression was found to be inversely associated to AKT pathway activation. Prognostic analysis showed that the prognosis is poorer for low CDK5RAP3 and UFM1 patients, than for high CDK5RAP3 and/or UFM1 (p<0.001) patients, and this expression pattern was an independent predictor for overall survival of GC. Coexpression of CDK5RAP3 and UFM1 combined with TNM staging can improve the accuracy of prognosis prediction for patients (p <0.001).

**Conclusions:**

It is confirmed in our findings that a combination of CDK5RAP3 and UFM1 can produce a more precise prediction model for GC patients’ survival.

## Introduction

As a well-known malignant tumor, gastric cancer leads to high lethality of patients worldwide, which makes it rolling as a leading cause to death. The latest epidemiological survey showed that gastric cancer ranks fifth and third global incidence and mortality rates, respectively, among malignant tumours. Globally, more than one million new gastric cancer cases are diagnosed every year, and approximately 800,000 people die of gastric cancer (1). At diagnosis and therapy, majority of patients are already at an advanced stage because of low specificity of early gastric cancer symptoms. Advanced gastric cancer patients have an unfavourable prognosis, with just around a 15% 5-year survival rate (2).. Accurate prognostic assessment helps to formulate reasonable treatment plans and follow-up plans. The TNM staging system is the foremost predictor for gastric cancer prognosis. However, even with the same TNM stage, the prognosis of patients is not the same. In 2014, data from the Gastric Cancer Genome Atlas Research Network confirmed the molecular heterogeneity of gastric cancer (3). Therefore, the prognostic evaluation of the biological potential of gastric tumours has attracted increased attention. It has vital theoretical and clinical significance for the prognostic evaluation of gastric cancer to explore the molecular markers for early identification of gastric cancer and the important role of molecular targeted therapy.

CDK5RAP3, known as C53, is an activation binding protein of cyclin-dependent kinase 5; it contains 506 amino acid residues and has a zinc-leucine zinc finger structure (4). CDK5RAP3 plays a key role in the formation and evolution of various malignant tumours (5). In our previous researches, we found that CDK5RAP3 can inhibit the phosphorylation of AKT in gastric cancer (6), thereby inhibiting the GSK-3β mediated phosphorylation, degrading β-catenin and acting as a tumour suppressor in the occurrence and progression of gastric cancer (7).

UFM1, a amall ubiquitin protein that contains 85 amino acids, was first discovered by Komatsu in 2004. UFM1 is first activated by UBA5 and is then converted into UFC1 and UFL1. UFL1 recognizes and helps UFM1 to bind the target protein. Finally, UFM1 processes and modifies the target protein to perform its biological vital activities. UFM1 and its modification system participate in different pathophysiological and biological processes, including the cell cycle, fatty acid β oxidation, cell survival, and hypoxia tolerance (8–10). Research has demonstrated that the development of breast cancer involves UFM1 (11). In previous studies, we found that UFM1 can also negatively regulate PI3K/AKT signalling by increasing the ubiquitination of PDK1 to inhibit the invasion and metastasis of gastric cancer (12).

The Akt-related signal transduction pathway is a complex signalling network mediated by growth factor receptors (GFRs) (13). Activation of this pathway suppresses cell apoptosis triggered by different stimuli, increases progression and proliferation of the cell cycle, participates in the neovascularization, plays an important role in the formation of tumours, and participates in invasion and metastasis of tumours (14–16). Thereby, we considered that CDK5RAP3 and UFM1 may play a coordinated role in inhibiting the gastric cancer invasion and metastasis. Although some studies have suggested that UFM1 binds to CDK5RAP3, the expression of the two proteins and their effects on the prolonged survival in gastric cancer have not been documented yet.

Therefore, we investigated the correlation between UFM1 and CDK5RAP3 expression and the prognosis of gastric cancer using public databases. We also detected the expression of the two indicators in 215 gastric cancer tissue samples using IHC, Western blotting and qPCR. To improve the accuracy of judging prognosis in gastric cancer, the relationship between expression of these two proteins and relevant clinical and pathological characteristics, as well as long-term survival in patients was analysed.

## Materials and methods

### Gastric cancer data sets

We searched the published gastric cancer gene expression database systematically, including those with complete clinical information and excluding those with no survival information. Finally, we gathered The Cancer Genome Atlas Stomach Adenocarcinoma (TCGA-STAD) cohorts and 17 Gene Expression Omnibus (GEO) cohorts of samples from patients with GC for this study (GSE54129, GSE65801, GSE35809, GSE51105, GSE13861, GSE27342, GSE29272, GSE63089, GSE19826, GSE79973, GSE13911, GSE51575, GSE118916, GSE122401, GSE130823, GSE15459, GSE66229) and the TCPA database for analysis. The original data were collected and downloaded from GEO (http://www.ncbi.nlm.nih.gov/geo/), TCGA (https://portal.gdc.cancer.gov/), and TCPA (https://www.tcpaportal.org/tcpa).

### Tissue specimens

The tissues in this study were selected from gastric adenocarcinoma tissue specimens of 215 patients undergoing radical gastrectomy for gastric cancer in our center from January 2013 to December 2014. All patients were newly diagnosed and before surgery they had not received chemotherapy or radiation treatment. The patients were pathologically confirmed to have gastric adenocarcinoma after surgery with comprehensive clinicopathological information. The data were analyzed retrospectively. This study was approved by the Fujian Medical University Union Hospital Ethics Committee and written permission was obtained from every relevant patient.

### Single-sample gene set enrichment analysis

We obtained 3 GFR gene sets (KRAS_SIGNALING_UP and AKT_UP. V1_DN and MTOR_UP. V1_DN) from C6 (oncogenic gene sets) of MSigDB (https://www.gsea-msigdb.org/). Using the R software package “GSVA” (gene set variation analysis for microarray and RNA-seq data), we scored each sample in the TCGA cohort by ssGSEA (method = “ssgsea”, ssgsea.norm = TRUE, verbose = TRUE).

### Unsupervised clustering

Unsupervised clustering methods (K-means) were used to classify the TCGA cohort into different clusters based on the enrichment of GFR pathways. The clustering factors were the ssGSEA scores of the three GFR gene sets. These scores were first converted to z scores to improve the accuracy of clustering. We determined the final number of clusters according to the algorithm provided by the R software package “NbClust”. Finally, the TCGA queue was accurately divided into 3 clusters defined as Cluster A, Cluster B, and Cluster C.

### GSEA

We performed GSEA on the TCGA and GEO datasets (GSE54129, GSE65801, GSE35809, and GSE51105). First, we used the mean ± standard deviation (SD) of the CDK5RAP3 expression value as the cut-off point to divide each data set into three groups: the group of high, moderates and low. Next, we compared the high and low expression group to obtain differentially expressed genes. Additionally, the R package “clusterProfiler” (v3.12.0)0 (https://guangchuangyu.github.io/software/clusterProfiler) was applied to perform GSEA on these differential genes. MSigDB provided us with all of the hallmark and oncogenic gene sets (https://www.gsea-msigdb.org/).

### Immunohistochemistry

Tumour specimens containing enough formalin-fixed and embedded by paraffin were sliced into 4-μm serial sections and mounted for immunohistochemical analysis on silane-coated glass slides. The sections were dewaxed, rehydrated, antigen repaired, blocked and then incubated with appropriate antibodies. The rabbit anti-human CDK5RAP3 (ab24189; 1:200; Abcam) or UFM1 (ab109305; 1:200; Abcam) antibody was used as the primary antibody.

### Immunohistochemical score

Two experienced pathologists independently assessed IHC-stained tissue slices and scored them based on the intensity of cell staining and the positive ratio of the stained tumour cells. The proportion and intensity of CDK5RAP3-positive and UFM1-positive cells in random selection visual areas were evaluated to indicate the protein expression level. The following were the staining score standards for CDK5RAP3 and UFM1: no staining was indicated by a score of 0; the light yellow was defined as mild staining with a score of 1; the yellowish brown was defined as moderate staining with a score of 2; the brown was defined as significant staining with a score of 3. The following were the proportional score standards for stained tumor cells: when less than or equal to 5 percent cells were positive, the score was 0; when the positive cells were range from 6 to 25 percent, the score was 1; when the positive cells were range from 26 to 50 percent, the score was 2; when the positive cells were greater than or equal to 50 percent, the score was 3. (**Figure S1**). The final score ranging from 0 to 9 for the expression of CDK5RAP3 and UFM1, was obtained by multiplying the staining score and proportional score. The low-expression group was defined as patients having a final score <4. The high-expression group included the remaining patients.

### Western blotting

We cut fresh soy-sized gastric cancer tissue and paracancerous tissue pieces into a shaking tube. Next, lysis solution was added (1 mg of tissue plus 6 µl of lysis solution). The lysis solution comprising RIPA lysis solution (Thermo Fisher Scientific, Waltham, MA, USA) + PMSF solvent + Cocktail (Roche, South San Francisco, CA, USA) was prepared (100:1:1). The tubes were then placed in the oscillator at 5 m/s for 30 s. Thereafter, the samples were subjected to shaking after 12000 rpm 4 times, followed by centrifugation for 5 min. The supernatant was then pipetted into a new EP tube. The protein concentration was measured by the BCA method, and the protein sample (loading volume per well 40 μg) was separated by 10% SDS-PAGE and transferred to a PVDF membrane. Subsequently, the membrane was blocked with 5% skim milk for 1 hour at room temperature. Next, the membrane was incubated with primary antibodies (CDK5RAP3, UFM1 and GAPDH) at 4°C overnight. After that, the membrane was washed with washing buffer (TBS-T) 3 times, 5 min each time, and then incubated with HRP secondary antibody (Cell Signaling Technology) for 1 h at room temperature. GAPDH was used as an internal control. Finally, the membrane was washed with TBS-T for 30 min and the protein bands were detected by enhanced chemiluminescence (Amersham Corporation, Arlington Heights, IL, USA). The following antibodies were used by Western blots: CDK5RAP3 (ab24189; 1:1000 dilution; Abcam, Cambridge, MA, USA), UFM1 (ab109305; 1:1000 dilution; Abcam, Cambridge, MA, USA), p-AKT (serine 473) (ab81283, 1:1000 dilution; Abcam, Cambridge, MA, USA) and GAPDH (#5174; 1:2000 dilution; Cell Signaling Technology).

### Total RNA extraction and qPCR

Total RNA from gastric cancer and paracancerous tissues was extracted using Invitrogen’s TRIzol kit according to the manufacturer’s instructions and used to obtain cDNA using Takara’s reverse transcription system. The copy numbers of GAPDH, CDK5RAP3 and UFM1 were detected using qPCR. The following were the detailed primer sequences:

CDK5RAP3 Forward primer: 5′-GCTGGTGGACAGAAGGCACT-3′

Reverse primer: 5′-TGTCCTGGATGGCAGCATTGA-3′

UFM1 Forward primer: 5′-GTCCCC AGCACACTAGAGGA-3′

Reverse primer: 5′-GGA AAAGAGCGGGAG AGAGT-3′

GAPDH Forward primer: 5′-GAAGGTGAAGGTCGG AGT-3′,

Reverse primer: 5′-GAAGATGGTGATGGGATTTC-3′

GAPDH was used as an internal reference, and the ΔΔCt method was used for analysis.

### Co-immunoprecipitation

Protein was extracted from stably transfected cells (HGC-27) overexpressing UFM1, and the BCA method was used to determine the protein concentration. A small amount of protein solution was saved and boiled with 2× SDS sample buffer and then frozen at -20°C for Western blot analysis. Next, an appropriate amount of UFM1 antibody was added to the remaining protein solution at a ratio of 100 µg of protein/1 µg antibody and incubated at 4°C with gentle shaking overnight. Protein A/G agarose beads (20 µl) were incubated at 4°C for 2–4 h and centrifuged at 4°C at 3000×g for 3 min. It discarded the supernatant and washed the agarose beads on 5 times with a buffer of 1 ml lysis. After the final removal of the supernatant, 20 µl of 2× SDS was added to the pellet, followed by boiling in water for 5 min. Finally, the CDK5RAP3 antibody was used for Western blot.

### Follow-up

According to the institutional follow-up protocol, qualified doctors monitored all patients by outpatient clinics, phone calls, emails, letters or visits. The first 2 years of follow-up were completed every 3 months. The next 3 years of follow-up were completed every 6 months. Then they were followed up annually until death or after 5 years. Most of the patients had undergone physical exams, laboratory tests, imageological examinations and annual gastroscopy. The time from operation to last follow-up or death was defined as the overall survival time. The follow-up rate of the whole group was 93.56%, and the median follow-up time was 57 months (range, 2–83 months).

### Statistical analysis

All statistical analyses were performed using the Social Science Statistical Software Package (SPSS) version 23.0 for Windows (IBM, Chicago, IL, USA) or R software (version 3.6.2). If not specified, the results were shown as percentages or means ± SD. As needed, the data were analysed by chi-square test, Fisher’s exact test or Student’s t test. The survival rate was evaluated by Kaplan-Meier method and log-rank test. The Cox proportional hazards model was used for univariate and multivariate prognostic analysis. Multivariate analysis was performed on factors with p<0.05 in univariate analysis. Statistical significance was indicated when the P value was less than 0.05. Pearson’s correlation or Spearman’s correlation was used to estimate the correlation coefficient (p <0.05). Additionally, the protein interaction network was constructed using GeneMANIA (http://www.genemania.org/). A receiver operating characteristic (ROC) curve and the area under the curve (AUC) were computed to assess discriminative ability.

## Results

### The CDK5RAP3 and UFM1 genes were co-downregulated in patients with a poor prognosis

**Figure 1:**
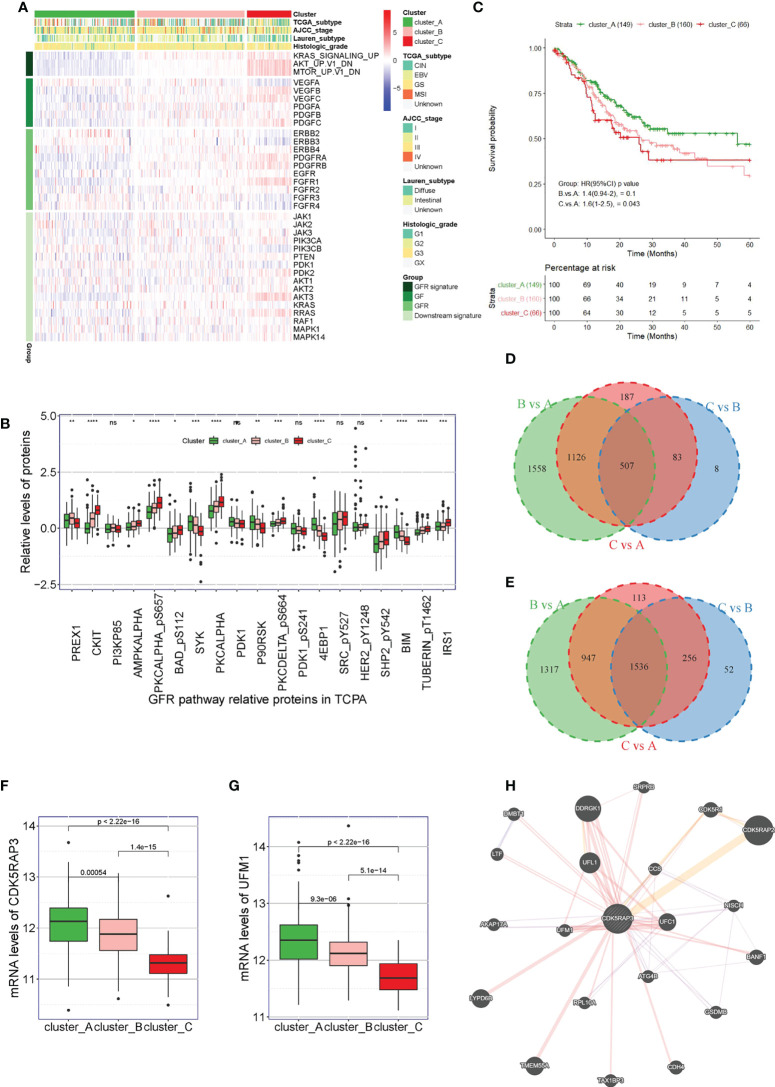
Correlation between UFM1 expression and GFR pathway and CDK5RAP3 expression. **(A)** Three hundred seventy-five patients in the TCGA cohort were divided into three groups (Cluster A, Cluster B and Cluster C) based on unsupervised analysis and hierarchical clustering of the ssGSEA scores of the three GFR gene sets. **(B)** Related proteins of the GFR pathway from the TCPA database. **(C)** Overall survival of patients in Cluster A, Cluster B and Cluster **(C, D)** Venn diagram of the common downregulated genes in clusters B *vs*. A, C *vs*. A and C *vs*. B. **(E)** Venn diagram of the common upregulated genes in clusters B *vs*. A, C *vs*. A and C *vs*. B. **(F)** mRNA levels of CDK5RAP3 in the patients in Cluster A, Cluster B and Cluster C. **(G)** mRNA levels of UMF1 in the patients in Cluster A, Cluster B, and Cluster C. **(H)** Analysis diagram of the protein interaction network (GeneMANIA). *p < 0.05; **p < 0.01; ***p < 0.001; ****p < 0.0001; ns, no significance.

First, we used unsupervised clustering methods to classify 375 tumour samples from The Cancer Genome Atlas (TCGA) database into three molecular subgroups (Cluster A, Cluster B, and Cluster C) based on the three characteristic pathways of GFRs: KRAS, AKT, and MTOR. The heat map showed that the downstream signalling pathway-related genes GFR signature, GF and GFR were inhibited in patients in Cluster A, while they were activated in patients in Cluster C (**Figure 1A**). By analysing the related proteins of the GFR pathway from The Cancer Proteome Atlas (TCPA) database, we observed that the GFR pathway-related proteins SYK, PDK1, P90RSK, 4EBP1, and BIM were found to be highly expressed in Cluster A, PREX was found to be highly expressed in Cluster B, and CKIT, AMPKALPHA, PKCALPHA_pS657, BAD_pS112, PKCALPHA, PACDELTA_pS664, SHP2542, TUBERIN_pT1462, and IRS1 were found to be highly expressed in Cluster C, with significant differences (**Figure 1B**). Survival analysis also indicated that the overall survival of the patients from Cluster C was lower than that of the patients from Cluster A (p = 0.043) (**Figure 1C**). To explore which genes played a key regulatory role in the GFR pathway, we compared the genetic changes in patients in Clusters B *vs*. A, C *vs*. A, and C *vs*. B. The Venn diagram showed that Clusters B *vs*. A, C *vs*. A and C *vs*. B had 507 common downregulated genes (**Figure 1D** and **Table S1**), and 1,536 common upregulated genes (**Figure 1E** and **Table S2**). Analysing the co-downregulated genes and CDK5RAP3-interacting proteins in the string database, we found that the CDK5RAP3 and UFM1 genes were included in the 507 common downregulated genes, and the mRNA levels of CDK5RAP3 and UFM1 in patients of category C were lower than those in patients of categories A and B. The log fold-change of CDK5RAP3 was -0.741 in Cluster C *vs*. Cluster A and -0.567 in Cluster C *vs*. Cluster B. The log fold-change of UFM1 was -0.636 in Cluster C *vs*. Cluster A and -0.423 in Cluster C *vs*. Cluster B. (**Figures 1F, G**). Additionally, an interaction was observed between the two proteins (**Figure 1H**).

### UFM1 and CDK5RAP3 were adversely linked to the AKT pathway

**Figure 2:**
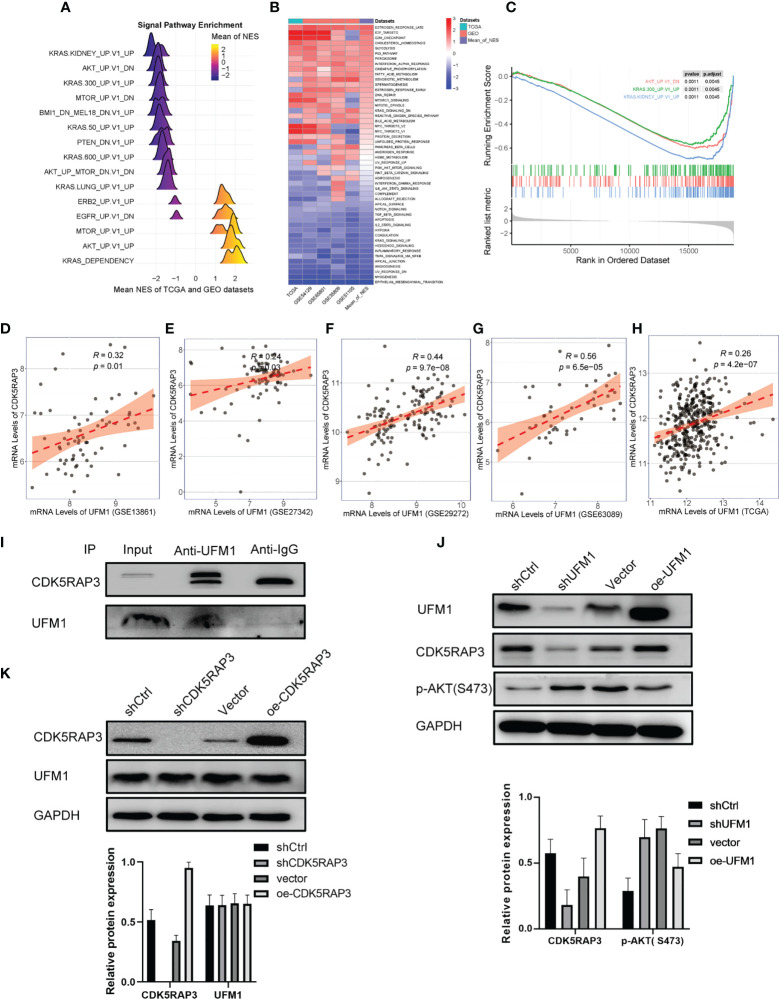
Pathway enrichment analysis of CDK5RAP3 and the correlation between UFM1 and CDK5RAP3 expression. **(A)** Mountain map of signal pathway enrichment. **(B)** Heatmap showing the activation status of the biological processes in different data sets. **(C)** GSEA enrichment analysis map for CDK5RAP3. **(D)** Pearson’s correlation of UFM1 and CDK5RAP3 expression in GSE13861. **(E)** Pearson’s correlation of UFM1 and CDK5RAP3 expression in GSE27342. **(F)** Pearson’s correlation of UFM1 and CDK5RAP3 expression in GSE29272. **(G)** Pearson’s correlation of UFM1 and CDK5RAP3 expression in GSE63089. **(H)** Pearson’s correlation of UFM1 and CDK5RAP3 expression in TCGA. **(I)** Co-immunoprecipitation using anti-UFM1 antibody to pull down the bait protein was used to detect the binding of CDK5RAP3 and UFM1 in the overexpressing UFM1 HGC-27 cells, with input as the positive control and anti-IgG as the negative control. **(J)** Western blot analysis of CDK5RAP3 and PAKT with knockdown and overexpression of UMF1 in the HGC cell line. **(K)** Western blot analysis of UFM1 with knockdown and overexpression of CDK5RAP3 in the HGC cell line.

We further performed pathway enrichment analysis of patients with high and low CDK5RAP3 expression in the TCGA and GEO databases. The mountain map, heat map and GSEA enrichment analysis map all indicated that CDK5RAP3 expression negatively correlated with AKT pathway activation (**Figures 2A–C**), a finding that was consistent with previous research results (6). Additionally, the correlation analysis of four GEO databases (GSE13861, GSE27342, GSE29272, GSE63089) and the TCGA database revealed that the expression levels of UFM1 and CDK5RAP3 were significantly correlated (**Figures 2D–H**). Co-IP experiments confirmed that UFM1 had a direct binding effect with CDK5RAP3 (**Figure 2I**). Therefore, we knocked down and overexpressed UMF1 and CDK5RAP3 in the HGC cell line to verify that UFM1 and CDK5RAP3 negatively correlated with AKT pathway activation. The results showed that knocking down UFM1 caused a decrease in CDK5RAP3 expression and reduced the inhibition of AKT phosphorylation, while the overexpression of UFM1 caused an increase in CDK5RAP3 to enhance the inhibition of AKT phosphorylation (**Figure 2J**). However, the UFM1 didn’t change when CDK5RAPS was knocked down or overexpressed (**Figure 2K**).

### The CDK5RAP3 and UFM1 expression was low in gastric cancer tissue

**Figure 3:**
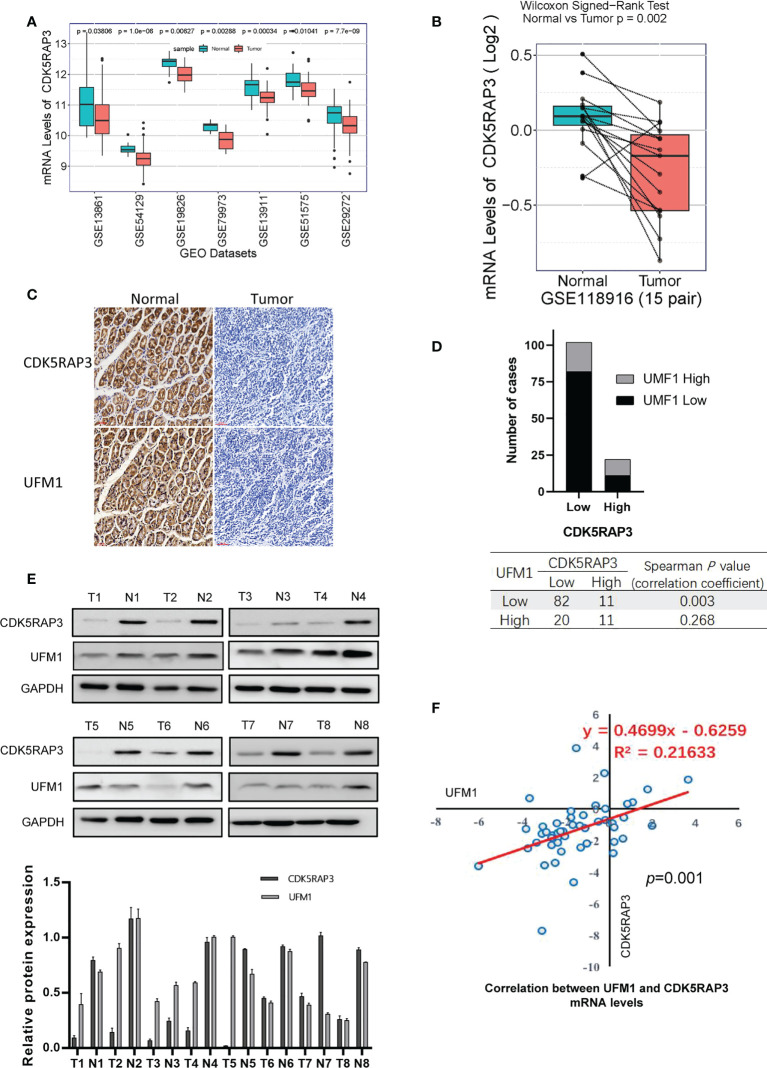
Expression levels of CDK5RAP3 and UFM1 in gastric cancer tissue. **(A)** mRNA levels of CDK5RAP3 in cancer and paracancerous tissues in 7 GEO databases. **(B)** mRNA levels of UMF1 in cancer and paracancerous tissues from 15 cases in GSE118916. **(C)** IHC staining of cancerous and paracancerous tissues from gastric cancer patient. **(D)** CDK5RAP3 was shown to be correlated with UFM1 expression. **(E)** Protein expression levels of CDK5RAP3 and UFM1 in gastric cancer and paracancerous tissues by Western blot. **(F)** Correlation of CDK5RAP3 and UFM1 mRNA expression in the gastric cancer tissues of 48 patients.

Analysis of 7 GEO databases (GSE13861, GSE54129, GSE19826, GSE79973, GSE13911, GSE51575, GSE29272) showed that CDK5RAP3 expression was low in gastric cancer (**Figure 3A**). CDK5RAP3 expression levels in cancerous and paracancerous tissues from 15 cases in GSE118916, 80 cases in GSE122401, and 47 cases in GSE130823 were found to be low (**Figure S2**), as was UFM1 expression in cancerous and paracancerous tissues from 15 patients in GSE118916 (**Figure 3B**). Furthermore, we used samples from the internal centre for verification. IHC staining of cancerous and paracancerous tissues from gastric cancer patient showed that CDK5RAP3 and UFM1 protein expression in cancerous samples were both lower than that in paracancerous (**Figure 3C**). IHC staining score was used to analyse CDK5RAP3 and UFM1 protein expression in paraffin-embedded gastric cancer samples from 124 patients. CDK5RAP3 was found to be lowly expressed in 102 patients (82.3%) and had high expressions in 22 patients (17.7%). The expression levels of UFM1 were found to be low in 93 patients (75. 5%) and high in 31 patients (25.0%). Spearman’s correlation analysis indicated that CDK5RAP3 and UFM1 expression was significantly correlated (**Figure 3D**). We also used Western blotting to detect CDK5RAP3 and UFM1 expression in the cancerous and paracancerous tissues of 43 gastric cancer patients (**Figure 3E**) and simultaneously detected the mRNA levels of CDK5RAP3 and UFM1 in the tumour tissues of 48 patients with gastric cancer. Pearson’s correlation analysis showed that the expression of the two mRNA levels was positively correlated (**Figure 3F**).

### Patients with low CDK5RAP3 and UFM1 expression had the worst prognosis

**Figure 4:**
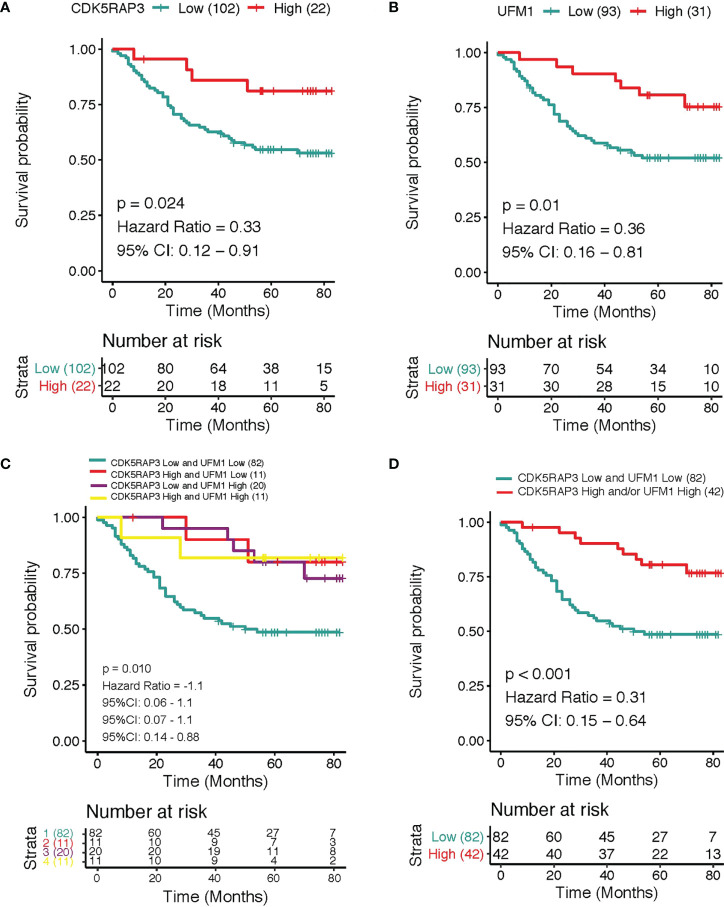
Overall survival according to different expression levels of CDK5RAP3 and UFM1. **(A)** Overall survival of gastric cancer patients with low and high expression of CDK5RAP3. **(B)** Overall survival of gastric cancer patients with low and high expression of UFM1. **(C)** Overall survival of gastric cancer patients with different co-expression of CDK5RAP3 and UFM1. **(D)** Comparison of the overall survival of CDK5RAP3 low and UFM1 low and CDK5RAP3 high and/or UFM1 high.

The overall survival was reduced dramatically in patients with low CDK5RAP3 expression compared with patients with high CDK5RAP3 in the 3 GEO databases (GSE13861, GSE15459 and GSE66229) and the TCGA database (**Figure S3**). Similarly, the overall survival rate was significantly worse among patients with low UFM1 than in patients with high UFM1 (**Figure S4**). In the GSE66229 database, the patients with low CDK5RAP3 expression had a significant lower disease-free survival rate than that of patients with high CDK5RAP3 expression, and the patients with low UFM1 expression also had a significant lower disease-free survival rate than those with high UFM1 expression (**Figure S5**). Regarding the internal centre data, the 3-year overall survival rate was 66.9% with median 57 months follow-up for the entire group. According to survival analyses, the 3-year cumulative overall survival rate of high CDK5RAP3 expression patients was significantly higher than that of low CDK5RAP3 patients (81.8% *vs*. 62.7%, p < 0.05, **Figure 4A**); those with low UFM1 expression exhibited a lower 3-year overall survival rate than patients with high UFM1 expression (58.1% and 90.3%, respectively; p 0.05; **Figure 4B**). We further analysed the prognostic value of the combination of CDK5RAP3 expression and UFM1 expression by Kaplan–Meier analysis. In comparison to the other groups of patients, patients with low expression levels of CDK5RAP3 and UFM1 had a poorer 3-year cumulative survival rate—only 54.9%—which was substantially below CDK5RAP3 high and/or UFM1 high expression patients (**Figure 4C**). After combing the groups, we found that patients with low CDK5RAP3 and UFM1 expression had a significantly worse prognosis than those with high CDK5RAP3 and/or UFM1 expression (88.1%) (p < 0.001; **Figure 4D**).

### TNM staging was an independent factor related to the low CDK5RAP3 and UFM1 expression

Analysis of factors associated with the expression of CDK5RAP3 and UFM1 in gastric cancer tissues showed that the CDK5RAP3 and UFM1 expression significantly correlated with BMI, lymph node metastasis, depth of invasion and pathological TNM stage (**Table 1**). Combining the low CDK5RAP3 and UFM1 expression, analysis of related factors showed that the low expression level of the two was related to tumour size, depth of invasion, lymph node metastasis and TNM staging (**Table 2**). BMI, tumour size and TNM staging were further included in the logistic regression model. The results of multivariate analysis suggested that TNM staging was an independent factor related to the low expression of CDK5RAP3 and UFM1 (I+II *vs*. III: 95% CI: 1.128–5.755, p = 0.023).

**Table 1:** The association of CDK5RAP3 and UFM1 expression in gastric cancer tissues with clinicopathological factors.

Variables	Total	CDK5RAP3 expression				UFM1 expression			
		low	high	*χ*2	p	low	high	*χ*2	p
**Gender**				0.076	0.783			0.341	0.5509
Male	98	79	19			73	25		
Female	26	23	3			20	6		
**Age (years)**				0.241	0.624			0.168	0.682
>60	87	75	12			68	19		
≤60	37	27	10			25	12		
**BMI (kg/m^2^)**				3.910	0.048			4.9119	0.027
>25	23	22	1			19	4		
≤ 25	101	80	21			74	27		
**Tumor size (cm)**				0.535	0.464			5.401	0.020
>5	48	41	7			34	14		
≤ 5	76	61	15			59	17		
**Tumor location**				4.403	0.111			4.191	0.123
Lower 1/3	55	46	9			37	18		
Middle 1/3	23	19	4			21	2		
Upper 1/3	46	37	9			35	11		
**Borrmann type**				0.209	0.647			0.945	0.331
I+II	27	20	7			22	5		
III+IV	97	82	15			71	26		
**Differentiation type**				4.075	0.044666			2.818	0.093
Undifferentiated	78	64	14			62	16		
Differentiated	46	38	8			31	15		
**pT stage**				8.678	0.003			8.264	0.004
T1+ T2	18	13	5			11	7		
T3+ T4	106	89	17			82	24		
**pN stage**				16.478	0.001			19.37774	0.000
N0	17	13	4			11	6		
N1	20	16	4			15	5		
N2	42	31	11			32	10		
N3	45	42	3			35	10		
**TNM stage**				11.373	0.001			11.2198	0.001
I+II	39	29	10			26	13		
III	85	73	12			67	18		
**Vessel invasion**				0.241	0.624			0.045	0.832
Negative	80	62	18			63	17		
Positive	44	40	4			30	14		

**Table 2:** The association of different CDK5RAP3 and UFM1 expression levels in gastric cancer tissues with clinicopathological factors.

Variables	Total	CDK5RAP3 and UFM1 low expression	CDK5RAP3 and/or UFM1 high expression	χ2	P
**Gender**					
Male	98	65	33	0.462	0.497
Female	26	17	9		
**Age (years)**					
>60	47	21	26	0.400	0.527
≤ 60	77	61	16		
**BMI (kg/m^2^)**					
>25	23	19	4	9.666	0.002
≤ 25	101	63	38		
**Tumor size (cm)**					
>5	48	31	17	4.972	0.026
≤5	76	51	25		
**Tumor location**					
Lower 1/3	55	33	22	3.848	0.146
Middle 1/3	23	18	5		
Upper 1/3	46	31	15		
**Borrmann type**					
I+II	27	20	7	1.146	0.284
III+IV	97	62	35		
**Differentiation type**					
Undifferentiated	78	54	24	3.045	0.081
Differentiated	46	28	18		
**pT stage**					
T1+ T2	18	9	9	7.965	0.005
T3+ T4	106	73	33		
**pN stage**					
N0	17	9	8	16.236	0.001
N1	20	12	8		
N2	42	27	15		
N3	45	34	11		
**TNM stage**					
I+II	39	20	19	9.422	0.002
III	85	62	23		
**Vessel invasion**					
Negative	80	54	26	0.001	0.981
Positive	44	28	16		

### The coexpression level of CDK5RAP3 and UFM1 was an independent prognostic factor for gastric cancer

Cox regression analyses were used to clarify the prognostic value of CDK5RAP3 and UFM1 expression. Based on the univariate analysis, overall survival was related to BMI, tumour size, TNM staging and combined CDK5RAP3 and UFM1 expression (**Table 3**). Multivariate analysis indicated that the coexpression level of CDK5RAP3 and UFM1, as well as TNM stage were both independent predictive variables for patient prognosis with gastric cancer (**Table 3**).

**Table 3:** Cox regression analysis of prognostic factor for gastric cancer.

Variable	Univariate Model				Reduced Multivariate Model			
	HR	95%CI		p	HR	95%CI		P
**Gender**				0.631				
Female	Ref							
Male	0.849	0.43	1.65	0.631				
**Age (years)**				0.960				
≤ 60	Ref							
> 60	0.985	0.53	1.80	0.960				
**BMI (kg/m^2^)**				0.020				0.156
≤ 25	Ref				Ref			
> 25	2.078	1.12	3.85	0.020	1.579	0.84	2.97	0.156
**Tumor size (cm)**				0.044				0.069
≤ 5	Ref				Ref			
> 5	1.758	1.02	3.05	0.044	1.685	0.96	2.95	0.069
**Tumor location**				0.121				
Lower 1/3	Ref							
Middle 1/3	1.246	0.56	2.78	0.590				
Upper 1/3	1.879	1.01	3.47	0.044				
**Borrmann type**				0.484				
I+ II	Ref							
III+IV	1.293	0.63	2.66	0.484				
**Differentiation type**				0.055				
Undifferentiated	Ref							
Differentiated	0.547	0.30	1.01	0.055				
**TNM stage**				0.001				0.003
I+II	Ref				Ref			
III	3.885	1.75	8.65	0.001	3.320	1.48	7.42	0.003
**Vessel invasion**				0.903				
Negative	Ref							
Positive	0.965	0.54	1.71	0.903				
**CDK5RAP3/UFM1 expression**				0.002				0.006
CDK5RAP3 low and UFM1 low	Ref				Ref			
CDK5RAP3 high and/or UFM1 high	0.312	0.15	0.64	0.002	0.357	0.17	0.74	0.006

### Combined expression of CDK5RAP3 and UFM1 to improve the accuracy of long-term prognosis evaluation

**Figure 5:**
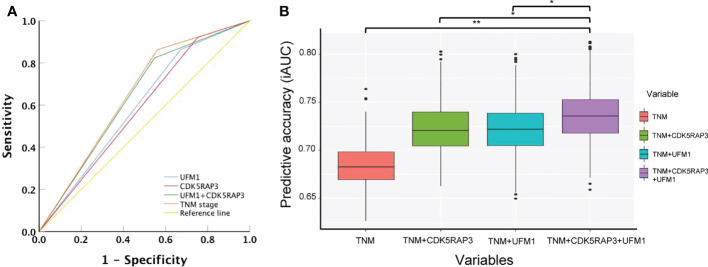
Combined CDK5RAP3 and UFM1 expression was used to evaluate the prognosis of patients with gastric cancer. **(A)** Receiver operating characteristic analysis of the predictive value. **(B)** Comparison of the prognostic prediction accuracy of patients among CDK5RAP3 or UFM1 combined with TNM staging, coexpression of CDK5RAP3 and UFM1 combined with TNM staging and separate TNM staging (A box plot depicts the forecast accuracy of the 5-year overall survival based on the iAUC with 1000× bootstrap resampling). *p < 0.05; **p < 0.01.

We compared the accuracy of CDK5RAP3 or UFM1 expression, as well as combined CDK5RAP3 and UFM1 expression and TNM staging, in predicting gastric cancer survival using ROC curve analysis. The combination expression of CDK5RAP3 and UFM1 was more accurate in predicting patient survival than either CDK5RAP3 or UFM1 expression on its own (AUC was 0.638, 0.584, and 0.596; 95% CI was 0.532–0.740, 0.473–0.688, and 0.490–0.702; p = 0.021, 0.172, and 0.104 for CDK5RAP3 + UFM1, CDK5RAP3 and UFM1 respectively). Additionally, combined CDK5RAP3 and UFM1 expression had a prognostic value that was similar to TNM staging (AUC: 0.651, 95% CI: 0.601–0.786, p = 0.001; **Figure 5A**). Furthermore, compared with CDK5RAP3 or UFM1 combined with or without TNM staging, the coexpression of CDK5RAP3 and UFM1 combined with TNM staging further improved the prognostic prediction accuracy of patients (p < 0.001, **Figure 5B**). Thus, the combination of CDK5RAP3 and UFM1 expression had a higher prognostic ability for overall survival in GC patients.You may insert up to 5 heading levels into your manuscript as can be seen in “Styles” tab of this template. These formatting styles are meant as a guide, as long as the heading levels are clear, Frontiers style will be applied during typesetting.

## Discussion

Gastric cancer remains the third leading cause of death in China despite improvements in diagnosis and therapy in recent years (5, 17). To better guide diagnosis and therapy, identifying specific biomarkers linked to gastric cancer prognosis may help improve the accuracy of gastric cancerprognostic assessment (18–20). Based on our previous study and an examination of public databases, this study found that the expression of the UFM1 and CDK5RAP3 genes are downregulated synchronously in gastric cancer patients with poor prognosis and that an interaction occurs between the UFM1 and CDK5RAP3 proteins. Therefore, we chose to evaluate UFM1 as a prognostic factor with CDK5RAP3.

GFRs and their abnormal signal transduction are important mechanisms of tumorigenesis and development, and they have become hot topics of research in recent years (10, 21). Many studies have shown that the abnormal function of growth factors and their receptors is an important cause of tumour occurrence and development. Such growth factor receptors have tyrosine kinase activity and can regulate the activity of downstream signalling pathways through phosphorylation (22, 23). The PI3K/Akt signalling pathway plays an important antiapoptotic role. Abnormalities in Akt-related signalling pathways are also associated with the occurrence of various tumours (13, 24). Therefore, we used public databases to search for proteins related to the GFR signalling pathway and CDK5RAP3 and attempted to identify biological prognostic indicators for gastric cancer. It was suggested that UFM1 was positively correlated with CDK5RAP3 and its low expression was associated with poorer prognosis of gastric cancer. Previous studies have shown that multiple proteins related to CDK5RAP3 and UFM1 and their modification systems (such as UFC1 and UFL1) are closely related (14, 15, 25). The correlation analysis of multiple public databases in this study also proved that the CDK5RAP3 and UFM1 expression were found to be substantially linked.

To date, few studies have investigated the combined expression levels of CDK5RAP3 and UFM1 and its prognostic significance in gastric cancer. Therefore, in patients with gastric cancer, we assessed the relationship between relevant clinicopathological parameters and overall survival by detecting the CDK5RAP3 and UFM1 expression levels. In univariate analysis, low CDK5RAP3 expression was linked to a poor prognosis, and high UFM1 expression was linked to a better survival rate in gastric cancer patients, indicating that both CDK5RAP3 and UFM1 play a tumour suppressor role in gastric cancer. Further analysis of related factors showed that the CDK5RAP3 and UFM1 coexpression was strongly linked to the invasive depth, lymph node metastasis and TNM stage, indicating that the two proteins are closely related to tumour invasion and migration in gastric cancer. Additionally, we found that the functions of CDK5RAP3 and UFM1 in gastric cancer were positively correlated. Patients with low CDK5RAP3 and UFM1 expression had the worst prognosis; if either of the two proteins showed high expression, patient’s prognosis was dramatically better. We considered that because CDK5RAP3 and UFM1 both played a role as tumour suppressor proteins, when one of the two proteins was highly expressed, the tumour suppressor effect in gastric cancer results in no difference in survival. When both proteins were expressed at a low level, the inhibition of the tumour was relieved, resulting in the poorest prognosis of all groups. Further analysis showed that the accuracy of prognostic analysis using CDK5RAP3 and UFM1 expression was closer to the accuracy of TNM staging prognostic analysis and higher than that of using CDK5RAP3 or UFM1 expression alone. Therefore, combination of the CDK5RAP3 and UFM1 expression can improve the capacity to forecast the survival outcomes of patients with gastric cancer.

The TNM staging system has been identified as a major prognostic factor for the gastric cancer. It’s also a valuable foundation for the formulation of gastric cancer treatment. However, differences in the prognosis of the same stage patients persist. In this study, we combined the coexpression of CDK5RAP3 and UFM1 with TNM staging for prognostic analysis. In comparison to the conventional TNM staging’s forecast accuracy, combining CDK5RAP3 and UFM1 expression with TNM greatly improved the accuracy of predicting gastric cancer patient survival. This finding indicated that the coexpression level of CDK5RAP3 and UFM1 could increase the accuracy of gastric cancer prognostic evaluation. Maybe it is possible to build a more precise model combining CDK5RAP3, UFM1 and TNM staging to predict 5-year survival of gastric cancer after surgery. It is worth exploring in the subsequent research. As a result, in clinical practice, the coexpression of CDK5RAP3 and UFM1 can be used in cooperation with TNM staging to effectively guide treatment and follow-up of patients with gastric cancer.

This study mainly explored the impact of the coexpression level of UFM1 and CDK5RAP3 on the clinicopathological parameters of gastric cancer patients and its prognostic significance, providing a preliminary basis for further research. Further investigation of how UFM1 and CDK5RAP3 regulate AKT pathway and whether CDK5RAP3 and UFM1 are associated with metastasis would be highly significant. Therefore, the elucidation of related mechanisms warrant further study.

## Data availability statement

The datasets presented in this study can be found in online repositories. The names of the repository/repositories and accession number(s) can be found below: https://www.ncbi.nlm.nih.gov/, TCGA-STAD, GSE54129, GSE65801, GSE35809, GSE51105, GSE13861, GSE27342, GSE29272, GSE63089, GSE19826, GSE79973, GSE13911, GSE51575, GSE118916, GSE122401, GSE130823, GSE15459, GSE66229 https://www.tcpaportal.org/tcpa, TCPA https://www.gsea-msigdb.org/, GSEA.

## Ethics statement

The studies involving human participants were reviewed and approved by Fujian Medical University Union Hospital Ethics Committee. The patients/participants provided their written informed consent to participate in this study. Written informed consent was obtained from the individual(s) for the publication of any potentially identifiable images or data included in this article.

## Author contributions

ML, L-LC, and J-XL conceived of the study; ML, N-ZL, and L-LC conducted the experiment and performed the major analysis; ML and N-ZL prepare the manuscript; J-XL, C-MH, C-HZ, PL, J-WX, J-BW, JL, Q-YC, Y-HL, Z-HP, X-YZ, and Y-XM helped to collect the data and revise the manuscript critically for important intellectual content; All authors contributed to the article and approved the submitted version.

## Funding

This study was funded by Construction Project of Fujian Province Minimally Invasive Medical Center (NO.[2021]662). Scientific and technological innovation joint capital projects of Fujian Province (No. 2018Y9008). Fujian Provincial Health Technology Project (2018-1-40). China Scholarship Council (202108350068).

## Acknowledgments

We are thankful to Ju-Li Lin, Hua-Long Zheng, Guang-Tan Lin and Fujian Medical University Union Hospital for managing the gastric cancer patient database.

## Conflict of interest

The authors declare that the research was conducted in the absence of any commercial or financial relationships that could be construed as a potential conflict of interest.

The handling editor DP declared a past collaboration with the author L-LC.

## Publisher’s note

All claims expressed in this article are solely those of the authors and do not necessarily represent those of their affiliated organizations, or those of the publisher, the editors and the reviewers. Any product that may be evaluated in this article, or claim that may be made by its manufacturer, is not guaranteed or endorsed by the publisher.
