# Supplementary material for: Down-regulated expression of CDK5RAP3 and UFM1 suggests a poor prognosis in gastric cancer patients
Source: Front Oncol. 2022 Oct 27;12:927751. doi: 10.3389/fonc.2022.927751 (PMC9647057; doi:10.3389/fonc.2022.927751)
Supplement: Supplementary file 3 [file DataSheet_3.zip › 927751 RAW DATA UPDATE/WB raw data/c53ufm1 WB instruction.pptx]

## Slide 1
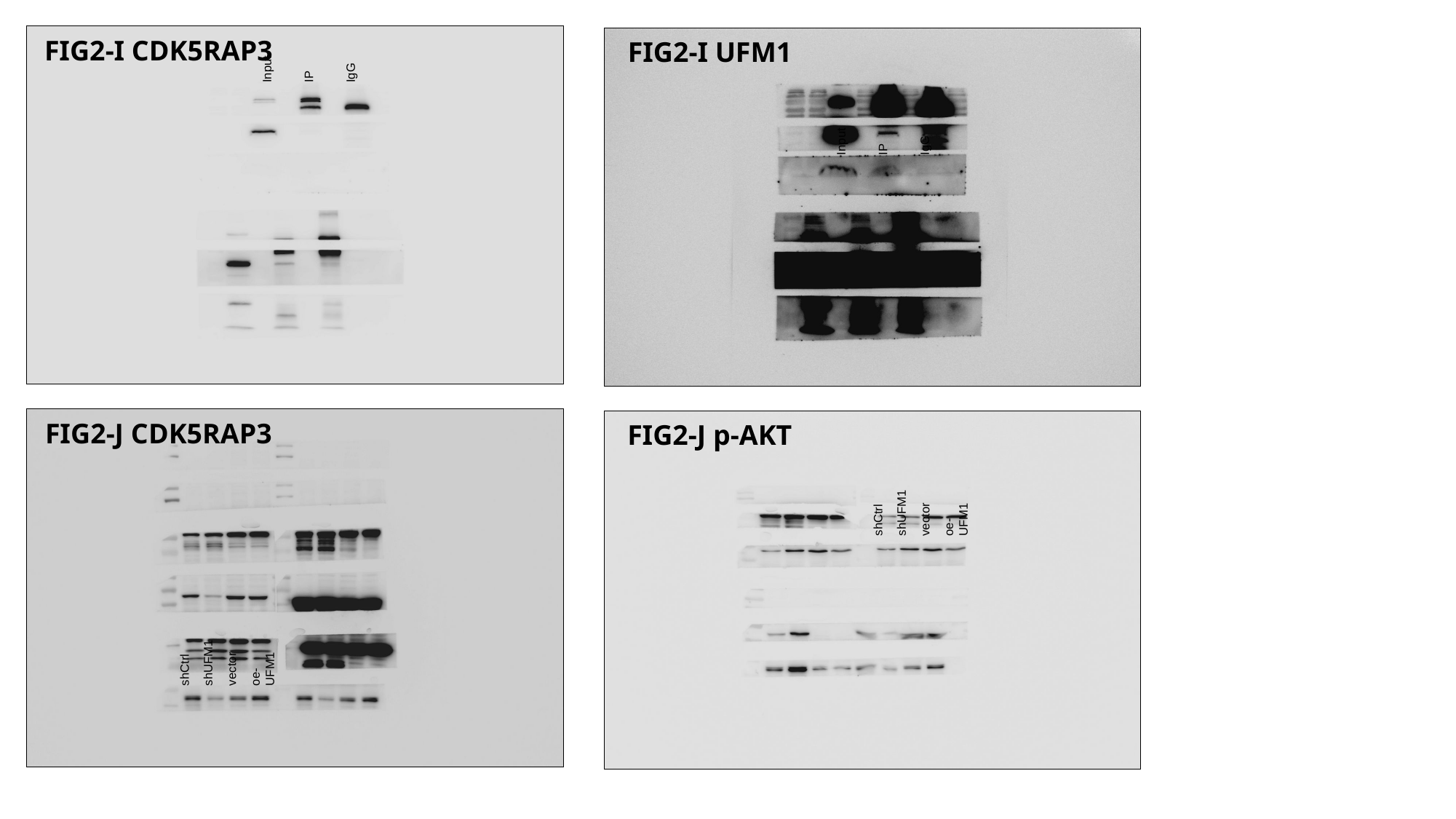

Input
IP
IgG
FIG2-I CDK5RAP3
FIG2-I UFM1
Input
IP
IgG
FIG2-J CDK5RAP3
FIG2-J p-AKT
shCtrl
shUFM1
vector
oe-UFM1
shCtrl
shUFM1
vector
oe-UFM1

## Slide 2
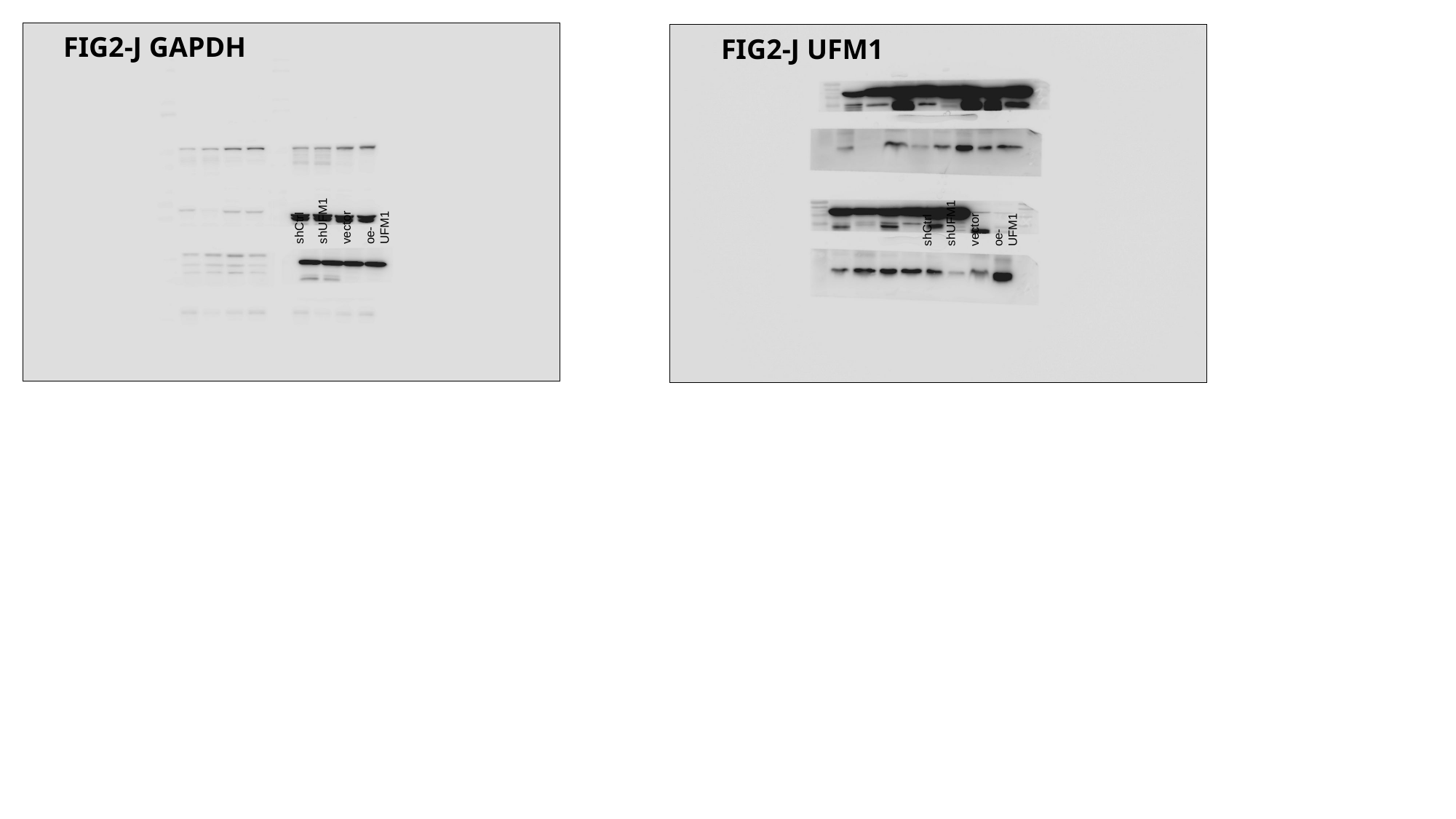

FIG2-J GAPDH
shCtrl
shUFM1
vector
oe-UFM1
FIG2-J UFM1
shCtrl
shUFM1
vector
oe-UFM1

## Slide 3
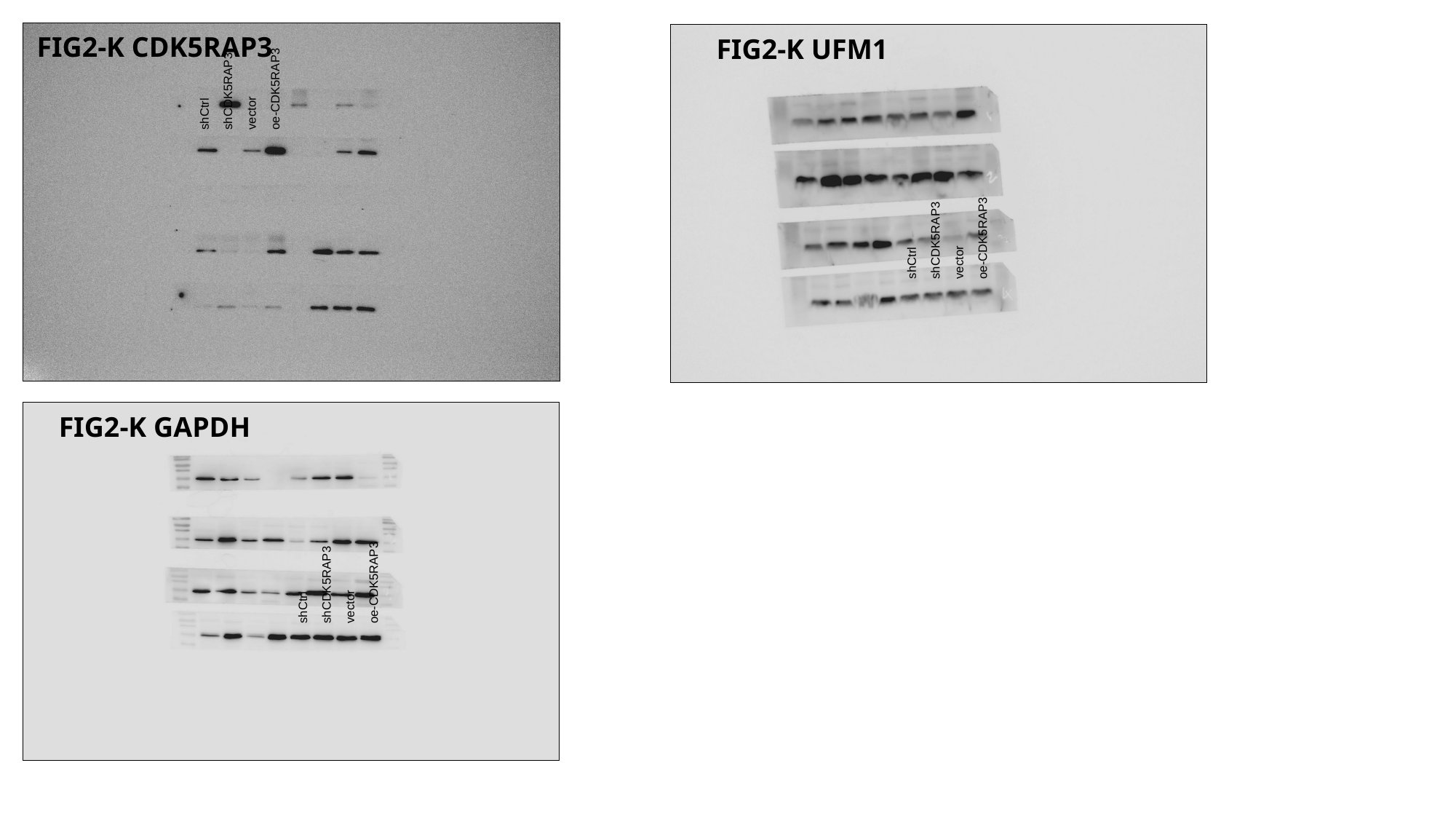

FIG2-K CDK5RAP3
shCtrl
shCDK5RAP3
vector
oe-CDK5RAP3
FIG2-K UFM1
shCtrl
shCDK5RAP3
vector
oe-CDK5RAP3
FIG2-K GAPDH
shCtrl
shCDK5RAP3
vector
oe-CDK5RAP3

## Slide 4
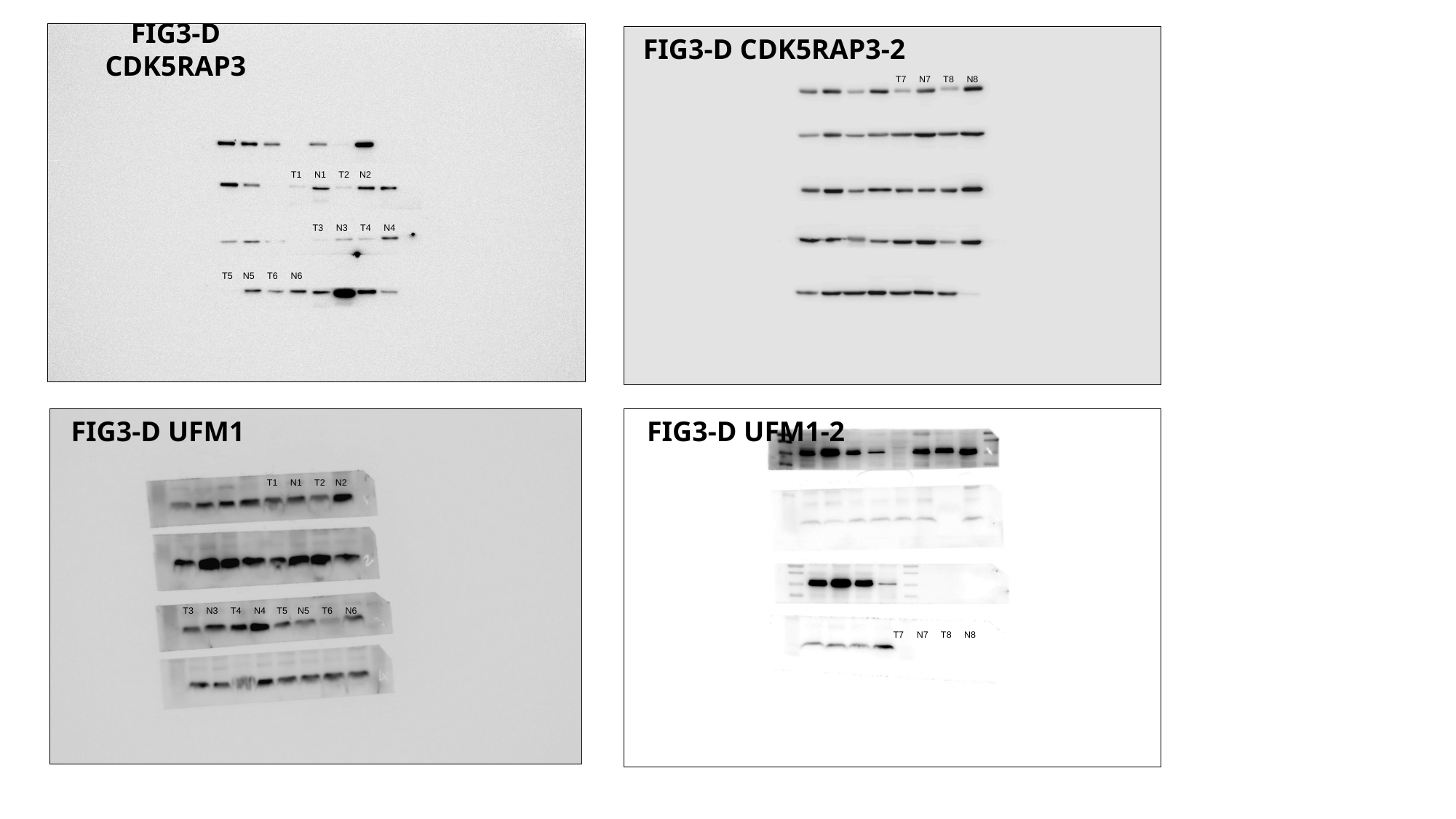

FIG3-D CDK5RAP3
FIG3-D CDK5RAP3-2
T7 N7 T8 N8
T1 N1 T2 N2
T3 N3 T4 N4
T5 N5 T6 N6
FIG3-D UFM1
FIG3-D UFM1-2
T1 N1 T2 N2
T3 N3 T4 N4
T5 N5 T6 N6
T7 N7 T8 N8

## Slide 5
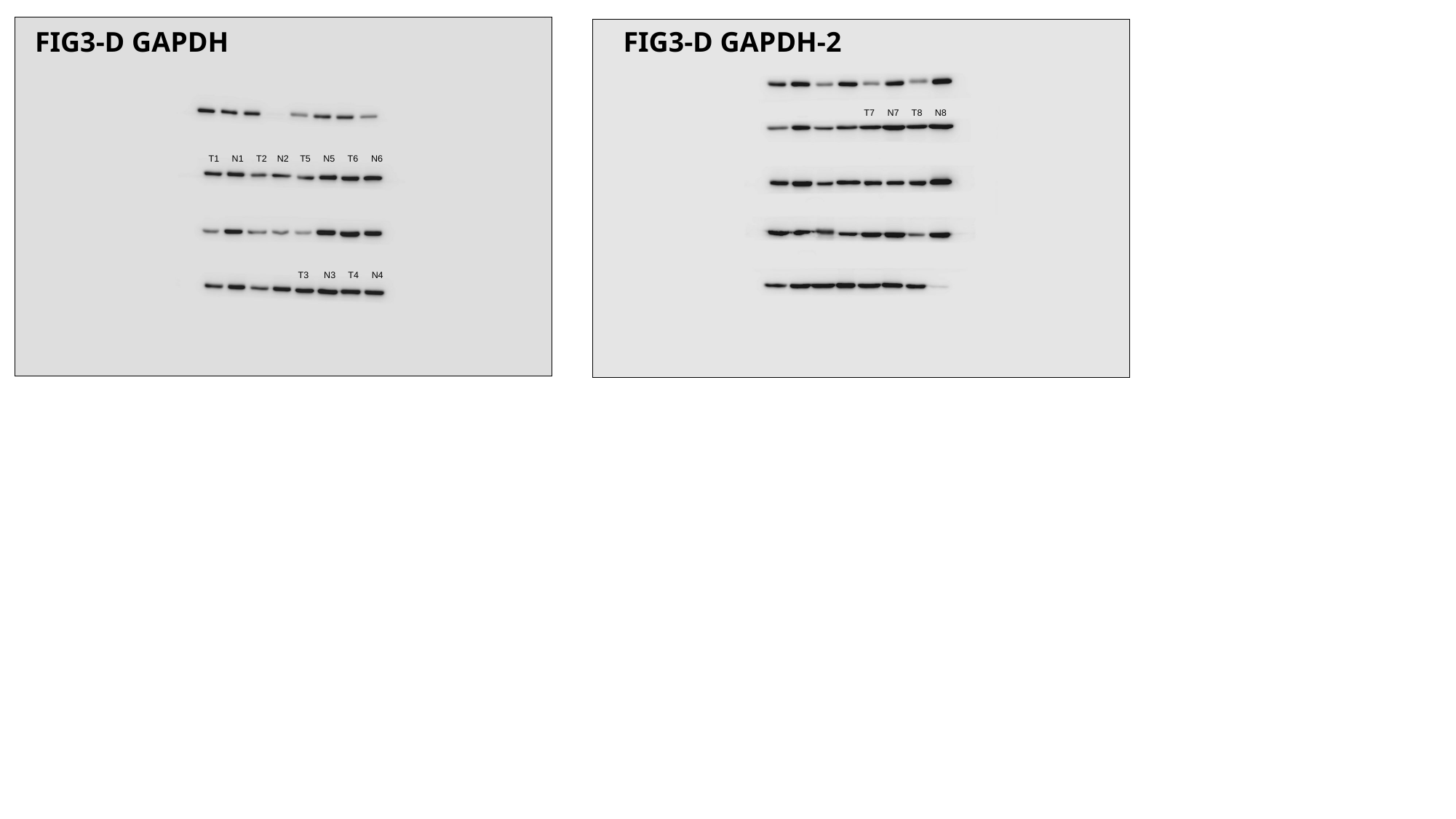

FIG3-D GAPDH
FIG3-D GAPDH-2
T7 N7 T8 N8
T1 N1 T2 N2
T5 N5 T6 N6
T3 N3 T4 N4
