# Supplementary figures and images for: Down-regulated expression of CDK5RAP3 and UFM1 suggests a poor prognosis in gastric cancer patients
Source: Front Oncol. 2022 Oct 27;12:927751. doi: 10.3389/fonc.2022.927751 (PMC9647057; doi:10.3389/fonc.2022.927751)

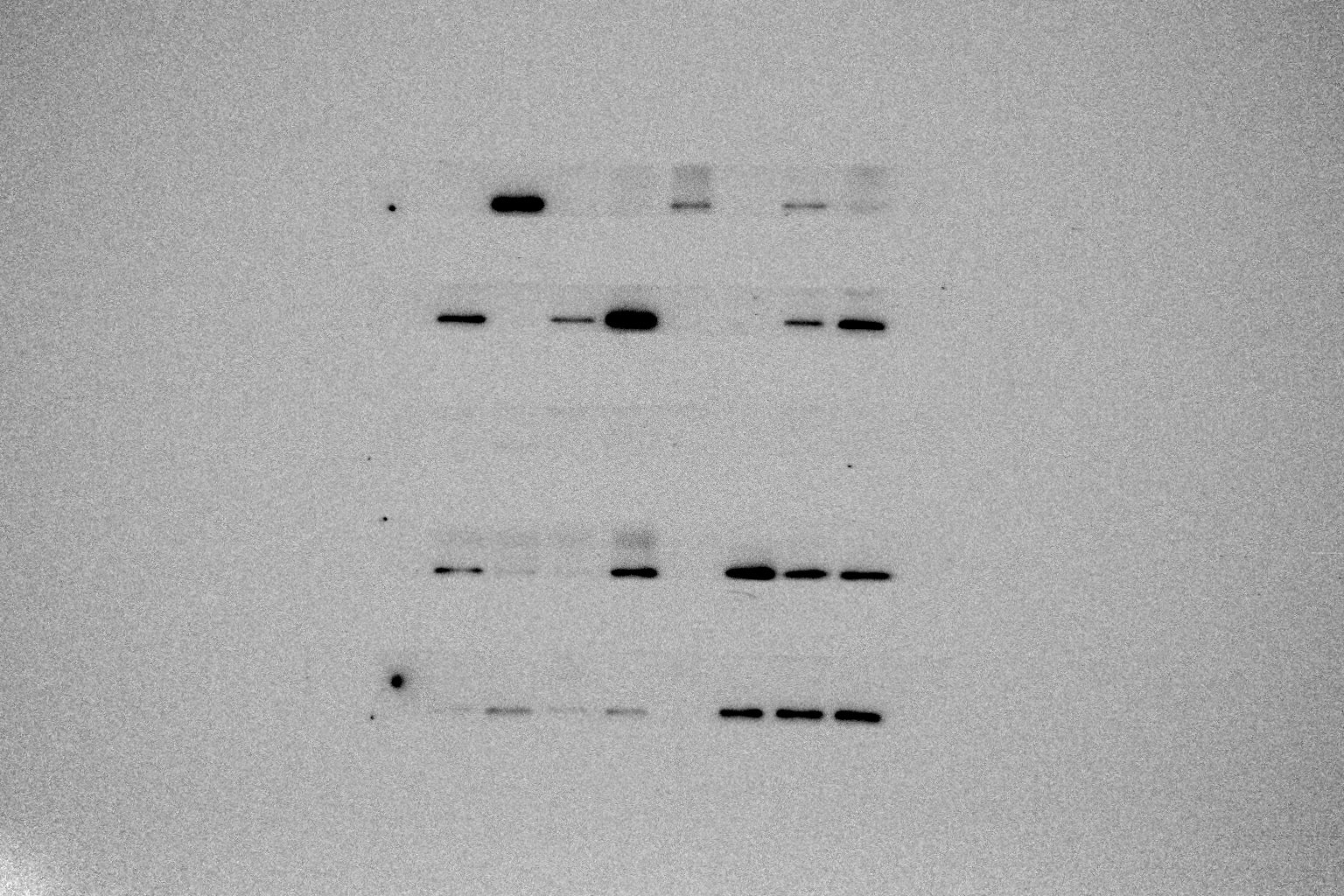

Supplement: Supplementary file 3 [file DataSheet_3.zip › 927751 RAW DATA UPDATE/WB raw data/FIG 2 K CDK5RAP3.tif]
