# Supplementary material for: Down-regulated expression of CDK5RAP3 and UFM1 suggests a poor prognosis in gastric cancer patients
Source: Front Oncol. 2022 Oct 27;12:927751. doi: 10.3389/fonc.2022.927751 (PMC9647057; doi:10.3389/fonc.2022.927751)
Supplement: Supplementary file 4 [file DataSheet_4.pdf]

Supplementary figures

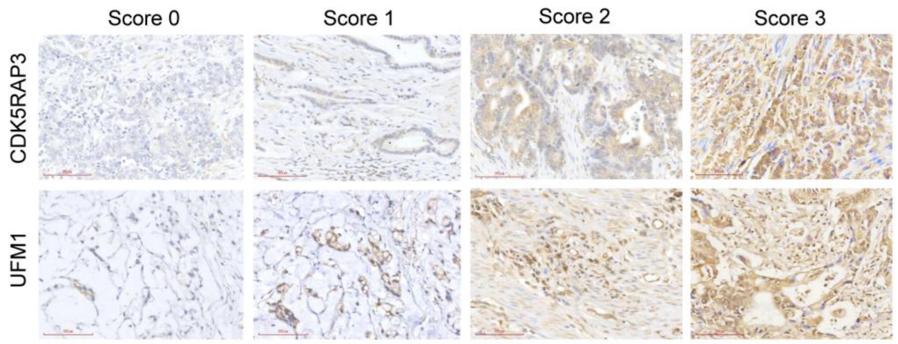

Figure S1. Immunohistochemical staining of CDK5RAP3 and UFM1 expression in gastric cancer tissues and the criteria for immunohistochemical scoring after a positive signal intensity.

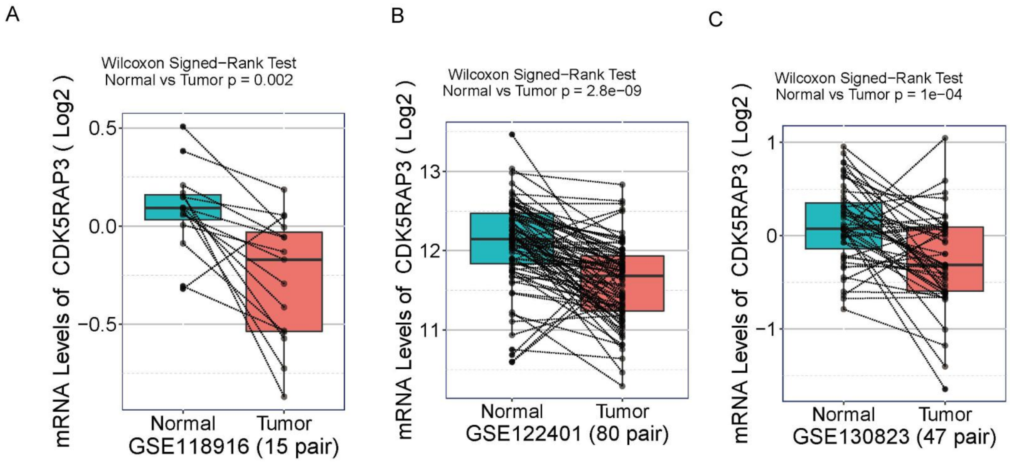

Figure S2. mRNA levels of CDK5RAP3 in cancer and paracancerous tissues in pairs. (A) in the GSE118916 database with 15 cases. (B) in the GSE122401 database with 80 cases. (C) in the GSE130823 database with 47 cases.

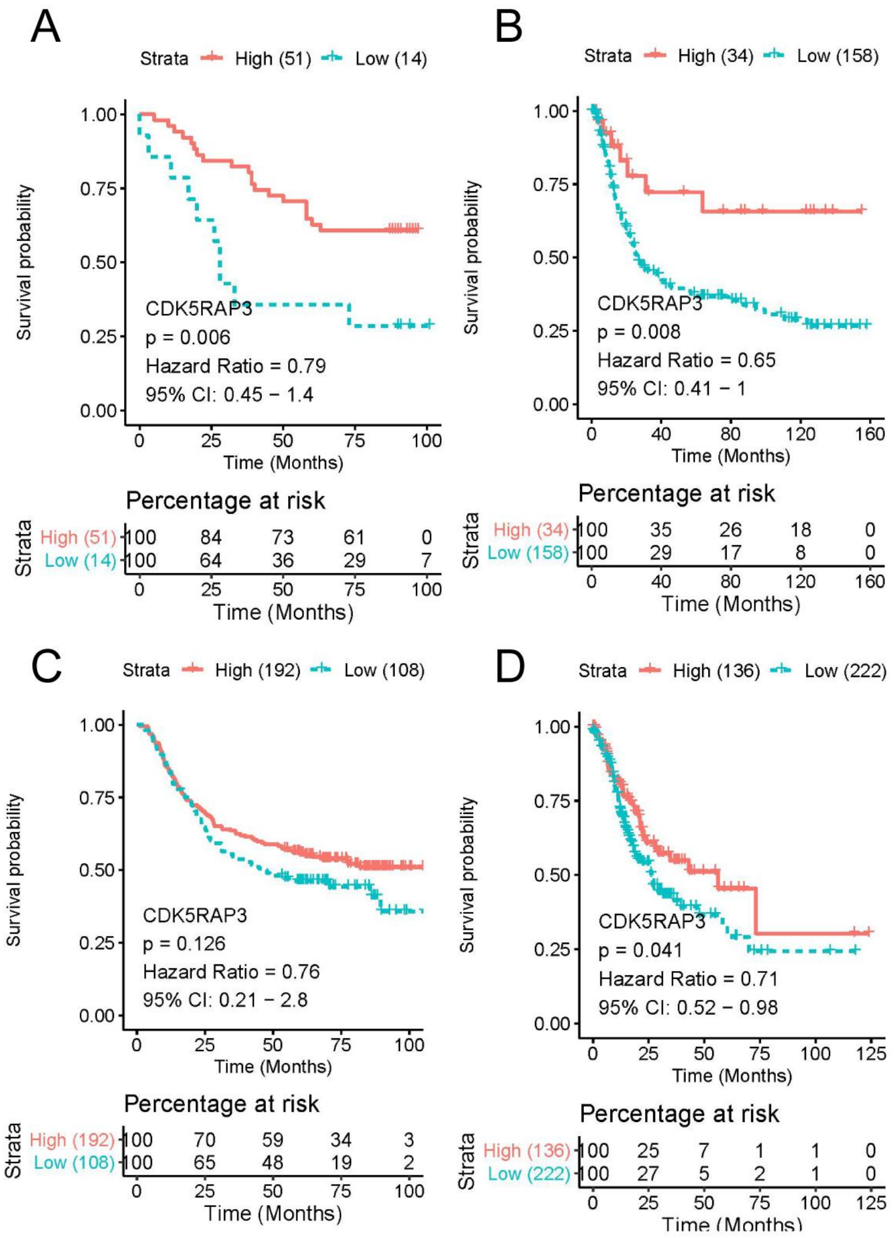

Figure S3. Overall survival of patients with low or high CDK5RAP3 expression. (A) in the GSE13861 database. (B) in the GSE15459 database. (C) in the GSE66229 database. (D) in the TCGA database.

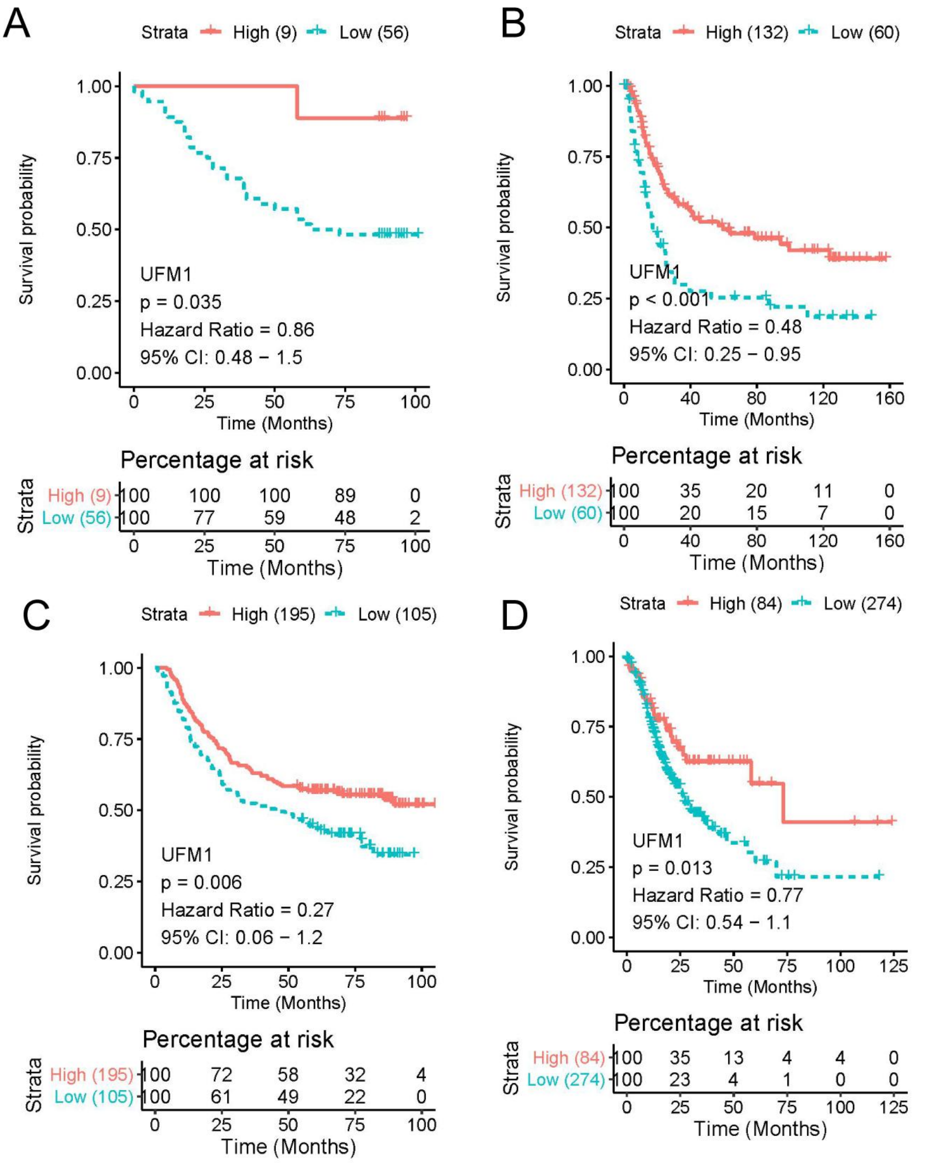

Figure S4. Overall survival of patients with low or high UFM1 expression. (A) in the GSE13861 database. (B) in the GSE15459 database. (C) in the GSE66229 database. (D) in the TCGA database.

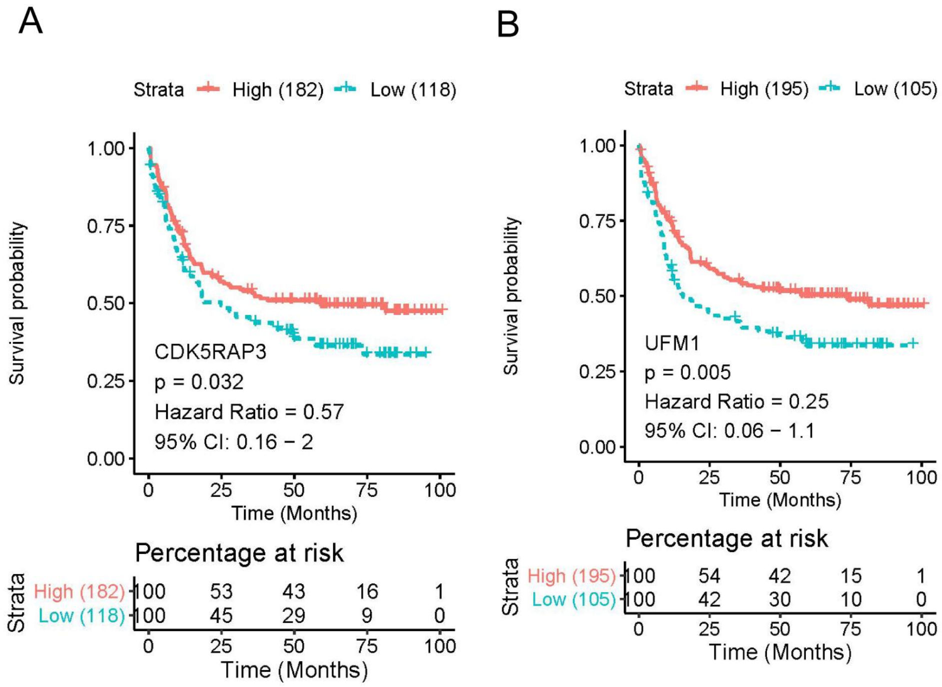

Figure S5. Disease-free survival of patients in the GSE66229 database. (A) with low or high CDK5RAP3 expression. (B) with low or high UFM1 expression.
